# Protocol  for the economic evaluation of LCS in Ireland: modelling costs, eligibility, and outcomes

**DOI:** 10.12688/hrbopenres.14126.1

**Published:** 2025-04-15

**Authors:** Tatiana Bezdenezhnykh, James O'Mahony, Benjamin Jacob, Deirdre Murray, Daniel Ryan, Jarushka Naidoo, Seamus Cotter, Alan Smith, Patrick Redmond

**Affiliations:** 1 General Practice, Royal College of Surgeons in Ireland, Dublin, Leinster, Ireland; 2 School of Economics, University College Dublin, Dublin, Leinster, Ireland; 3 National Cancer Registry, Cork, County Cork, Ireland; 4 Beaumont Hospital, Dublin, Leinster, Ireland; 5 Irish Lung Cancer Community, Dublin, Ireland; 6 National Screening Service, County Dublin, County Dublin, Ireland

**Keywords:** Lung cancer, Cost analysis, Cost-Effectiveness Analysis, Risk Assessment, Screening eligibility

## Abstract

**Background:**

Lung cancer (LC) is the leading cause of cancer death in Ireland, yet no national screening programme exists. While low-dose computed tomography (LDCT) screening reduces lung cancer mortality by approximately 20% in high-risk populations, its cost-effectiveness in Ireland remains uncertain. Evidence on the economic burden of lung cancer care and the feasibility of screening is needed to support policy decisions.

**Aim:**

This research programme will evaluate the economic impact of lung cancer care in Ireland and assess the cost-effectiveness of LDCT screening. By integrating screening eligibility modelling, stage-specific cost analysis, and economic evaluation, the study aims to generate evidence to support resource allocation and policy development.

**Methods:**

The programme consists of three interlinked work packages. First, screening eligibility will be estimated using a dynamic Markov model that integrates demographic data from the Central Statistics Office (CSO), population projections, and smoking history data from Eurobarometer. Second, a stage-specific cost analysis will be conducted using a discrete event simulation (DES) model informed by data from the National Cancer Registry Ireland (NCRI), the Healthcare Pricing Office (HPO), and other healthcare reimbursement sources. Third, a cost-effectiveness analysis will adapt a UK-based LC natural history model (Snowsill, 2018) to evaluate alternative screening strategies, incorporating Irish-specific costs, clinical outcomes, and quality-adjusted life-years (QALYs)

**Results and Implications:**

This programme will generate evidence to inform the design of a cost-effective LCS programme in Ireland. Findings will guide healthcare planning, optimise screening strategies, and support sustainable policy decisions.

## Introduction

### Lung Cancer: A Public Health Burden

In Ireland, lung cancer (LC) remains a leading cause of cancer-related deaths, with no organised national lung cancer screening (LCS) programme currently in place ^1^. Unlike some other cancers where survival has improved due to advances in early detection and treatment, LC continues to have poor survival outcomes, primarily due to late-stage diagnosis. The five-year survival rate in Ireland remains below 20%, reflecting the need for improved early detection strategies ^2^.

Despite strong international evidence supporting the effectiveness of low-dose computed tomography (LDCT) screening in reducing LC mortality, Ireland has yet to implement a national screening programme. Several barriers, including uncertainties surrounding cost-effectiveness, healthcare capacity, and resource allocation, have delayed policy action. Currently, LC is diagnosed predominantly through symptomatic presentation, which often occurs at an advanced stage when treatment options are more limited, costly, and less effective ^3^.

Countries such as the United States, United Kingdom, Australia, China, Portugal, and Hungary have introduced or piloted LDCT-based LCS programmes following the results of major trials, including the National Lung Screening Trial (NLST) and the Dutch-Belgian NELSON study ^4, 5^. However, the feasibility and cost-effectiveness of such programmes depend on national healthcare structures, smoking prevalence, participation rates, and economic considerations. These factors remain underexplored in Ireland, creating a critical gap in evidence to inform policy decisions. This research programme aims to address these gaps by conducting a comprehensive economic evaluation of LCS, integrating screening eligibility modelling, stage-specific cost analysis, and cost-effectiveness assessment.

### Epidemiology and Mortality Trends in Ireland

LC trends in Ireland differ markedly between men and women. While LC mortality rates in Irish men have declined significantly since the mid-1980s, rates among women have remained stable or increased. In 2012, Ireland had the eighth lowest LC mortality rate among men in Europe but the fifth highest among women ^1^. This divergence reflects historical smoking trends, where smoking prevalence among women increased later than in men, leading to a delayed but rising burden of lung cancer.

Projections suggest that these patterns will persist. Between 2015 and 2045, the age-standardised incidence rate of LC in Ireland is expected to decrease by 16% in males but increase by 29% in females ^2^. This shift has significant implications for healthcare planning, as the growing burden of LC among women may lead to increased demand for diagnostic and treatment services. Without effective early detection measures, the rising incidence will contribute to further strain on the Irish healthcare system.

### Economic Burden of LC in Ireland

LC care is among the most resource-intensive areas of oncology, with treatment costs increasing substantially for patients diagnosed at an advanced stage. The introduction of novel therapies, particularly immunotherapy and targeted treatments, has improved patient outcomes but has also substantially increased costs ^6, 7^. Late-stage LC is associated with prolonged hospital stays and intensive treatment regimens, placing further pressure on healthcare budgets. According to the NCRI, in 2016, lung cancer was the leading cancer type with health service costs attributable to modifiable risk factors, totalling approximately €62 million, all of which was linked to smoking ^8^.

Studies from other healthcare systems suggest that earlier detection through LDCT screening may reduce overall treatment costs by shifting diagnoses to earlier, more treatable stages. However, there is a lack of Ireland-specific data on the economic burden of LC by stage at diagnosis. Without such evidence, it is difficult for policymakers to determine the potential financial impact of a national screening programme and to allocate resources efficiently. Understanding the cost implications of LC treatment at different stages of disease progression is therefore a critical component of this research.

### The Importance of Early Detection

Early detection is the most effective strategy for improving LC survival. LDCT screening has been shown to reduce LC mortality by approximately 20–24% among high-risk populations ^9^. The NLST reported a 20% reduction in LC mortality with LDCT screening compared to no screening, while the NELSON trial demonstrated a 24% reduction in men and up to 33% in women ^10, 11^. In response to this growing body of evidence, the European Commission’s 2022 Council Recommendations on Cancer Screening identified LC as a priority for early detection, encouraging member states to develop risk-based screening approaches ^9^.

Economic evaluations from several countries, including the United Kingdom, China, Australia, and Portugal, indicate that LDCT screening is likely to be cost-effective, particularly for individuals aged 55–75 with a smoking history of at least 20 pack-years ^12– 16^. However, implementing a screening programme is not without challenges. LDCT screening introduces additional costs related to follow-up investigations, management of incidental findings, and the risk of overdiagnosis. Designing an efficient and sustainable screening programme requires a balance between clinical benefit and economic feasibility, ensuring that screening efforts are targeted towards those who will benefit most.

According to European Commission Country Cancer Profiles, Ireland has the second-highest rate of new cancer diagnoses in the EU, suggesting that the cost of cancer care is set to rise ^17^. Without a coordinated screening programme, Ireland risks failing to achieve the reductions in LC mortality observed in other countries. However, before implementation, critical evidence is needed to evaluate the financial and healthcare implications of screening within the Irish context.

### Key Evidence Gaps in Ireland

Despite the growing international consensus on the benefits of LCS, several key knowledge gaps hinder policy development in Ireland. The first major gap concerns the size of the high-risk population eligible for LDCT screening. While national smoking prevalence data exist, there is a lack of detailed pack-year history estimates, making it difficult to determine how many individuals would qualify for screening. Additionally, expected participation rates in an Irish LCS programme are unknown, introducing uncertainty into any cost-effectiveness projections.

The second gap relates to the economic impact of screening. No study has assessed the stage-specific costs of LC care in Ireland or estimated the potential cost savings from earlier detection. Understanding the total financial implications of implementing a national screening programme requires detailed cost data, including screening costs, follow-up procedures, and treatment expenses by disease stage.

Finally, Ireland, like many countries, has limited healthcare resources. A cost-effectiveness study can show whether implementing LCS is a good use of public funds compared to other healthcare interventions. By demonstrating who should be screened, the expected costs, and long-term benefits, it can guide policy decisions, secure funding, and ensure the program is both effective and financially sustainable. Cost-effectiveness analysis will inform a budget impact analysis (BIA), which estimates how much funding the Irish healthcare system (HSE) would need to roll out LCS nationwide.

### Aim and objectives

The overarching aim of this research programme is to conduct a comprehensive economic evaluation of LCS in Ireland, integrating screening eligibility modelling, stage-specific cost analysis, and cost-effectiveness assessment. This study will provide evidence to support policy decisions on the feasibility and design of a national screening programme.

The specific objectives are:

1.   To estimate the number of individuals eligible for LCS in Ireland, using demographic and smoking history data to model eligibility and participation rates.

2.   To quantify the stage-specific healthcare costs of lung cancer, capturing costs associated with treatment, follow-up care, and end-of-life management.

3.   To evaluate the cost-effectiveness of different LCS strategies, considering alternative screening frequencies, eligibility criteria, and resource implications.

## Methods

### Study design

This study is comprised of three interlinked work packages: (1) screening eligibility estimation, (2) stage-specific cost analysis, and (3) cost-effectiveness analysis (CEA). The study follows guidelines for economic evaluations and adheres to ISPOR-SMDM best practices for modelling studies, where relevant ^18, 19^. The analysis is conducted from the healthcare payer perspective (Health Service Executive, HSE), capturing direct medical costs associated with LCS, diagnosis, and treatment.

### Work Package 1: Estimating the Eligible Population for Screening

This work package will estimate the number of individuals eligible for LDCT screening in Ireland based on smoking history, demographic trends and population projections. The analysis will focus on individuals aged 55–74 years with a smoking history of 15 and 20 pack-years, the risk group typically recommended for LCS.

***Data sources and Inclusion criteria***

Three key publicly available datasets will be used:

•   Census of Population 2022 (CSO): Provides age- and sex-stratified demographic data for the Republic of Ireland ^20^.

•   CSO Population Projections (2022–2057): Enables long-term forecasting of screening demand ^21^.

•   Eurobarometer 87.1 (2017): Provides individual-level data on smoking status, smoking duration, and intensity (pack-years), allowing estimation of the high-risk population meeting LDCT eligibility criteria ^22^.

Individuals will be considered eligible if they meet the given pack-year threshold, calculated using smoking duration and intensity data from Eurobarometer. Exclusion criteria include never smokers and individuals outside the eligible age range.

***Modelling approach***

Using pack-year distributions from Eurobarometer data and Census 2022 data on smoking prevalence, the model will determine the proportion of individuals who exceed the eligibility threshold for screening in the base year (2022). In addition, population forecasts from the Central Statistics Office (CSO) (2022–2057) will be integrated to estimate the long-term demand for LCS in Ireland. A dynamic Markov-based population model will be used to estimate the number of high-risk individuals eligible for LDCT screening over time. The model will simulate the evolution of smoking behaviours, incorporating rates of smoking initiation, cessation, and relapse to reflect changes in the distribution of current and former smokers.

To ensure accuracy, the Markov model will be calibrated using historical smoking prevalence trends and validated against published Irish smoking data. This approach will allow for realistic projections of the future screening-eligible population, supporting evidence-based planning for LCS implementation.

### Work Package 2: Estimating Stage-Specific Costs of LC Care

This work package will estimate direct medical costs associated with LC care, stratified by disease stage (IA–IV) and treatment phase.

***Costing perspective and Data sources***

The cost analysis will adopt the healthcare payer perspective (Health Service Executive, HSE) and will focus on direct medical costs incurred within the public healthcare system. All costs will be adjusted to 2023 Euro values using the Irish consumer price index (CPI) for healthcare to ensure comparability with current economic conditions. Where necessary, missing cost components will be estimated using published unit cost data and validated through Delphi survey.

Data will be sourced from:

•   National Cancer Registry Ireland (NCRI): Provides data on incidence, stage distribution, survival, and treatment patterns.

•   Healthcare Pricing Office (HPO): Supplies cost estimates for hospital admissions, outpatient care, and procedures, mapped to Diagnosis-Related Groups (DRGs).

•   Hospital In-Patient Enquiry (HIPE) database: Provides hospital resource utilisation data, including length of stay and procedure frequencies.

•   Pharmaceutical Reimbursement Service (PCRS): Provides cost data for systemic therapies, including chemotherapy, immunotherapy, and targeted treatments.

***Costing methodology***

A discrete event simulation (DES) model will be used to estimate the stage-specific costs of LC care in Ireland ^23, 24^. The model will simulate a cohort of LC patients, tracking their diagnosis, treatment pathways, and healthcare utilisation over time. Costs will be assigned based on the stage at diagnosis (I–IV) and categorised according to the phase of care. The initial treatment phase will include diagnostic investigations, surgical procedures, systemic therapies, and radiotherapy. The surveillance and follow-up phase will incorporate costs associated with routine imaging, outpatient consultations, and disease monitoring. For patients with advanced or relapsed disease, the model will account for the costs of additional treatment lines, palliative care, and supportive interventions.

### Work Package 3: Cost-Effectiveness Analysis (CEA) of LCS

The CEA will compare LDCT screening to no screening, evaluating different screening strategies based on frequency (annual, biennial, one-time) and eligibility criteria (age, smoking history, risk thresholds).

***Model structure and inputs***

The CEA will use a DES model, adapted from an existing UK-based LC natural history model ^25^. Key model components include:

- Demographic inputs: Irish population structure (Census 2022, CSO projections).
- Epidemiological parameters: LC incidence, mortality, and stage distribution (NCRI).
- Screening effectiveness: Mortality reduction estimates from NLST (20%) and NELSON (24%) trials.
- Healthcare costs: Stage-specific treatment costs (from Work Package 2).
- Health outcomes: Life-years gained (LYG) and quality-adjusted life-years (QALYs), derived using Irish EQ-5D-5L utility weights (Hobbins et al., 2018).
- Screening-related harms: False positives, overdiagnosis, and procedure-related complications, drawn from published meta-analyses ^26, 27^.

The model will simulate a cohort of high-risk individuals undergoing screening, tracking LC incidence, stage shifts due to earlier detection, and treatment costs. Screening strategies will be compared based on frequency (annual, biennial, one-time) and eligibility criteria (age cut-offs, smoking history, risk stratification).

***Economic analysis and Uncertainty assessment***

The economic evaluation will estimate the incremental cost-effectiveness ratios (ICERs) for each LCS strategy, comparing them against a no-screening baseline. These ICERs will be assessed in relation to Ireland’s cost-effectiveness threshold of €45,000 per quality-adjusted life-year (QALY), as defined by the Health Information and Quality Authority (HIQA) ^28^. The analysis will adhere to CHEERS, ensuring transparency and methodological consistency ^18^.

To assess uncertainty in the model outputs, both probabilistic and deterministic sensitivity analyses will be conducted. A probabilistic sensitivity analysis (PSA) will incorporate uncertainty in key model parameters, using Monte Carlo simulations to generate cost-effectiveness acceptability curves (CEACs) (ref). These curves will illustrate the probability that different screening strategies remain cost-effective at varying willingness-to-pay thresholds. Simulations will assess parameter uncertainty, assuming gamma distributions for costs, beta distributions for probabilities, and log-normal distributions for relative risks. In addition, a deterministic one-way sensitivity analysis (DSA) will explore the impact of individual parameter variations, such as screening uptake rates, treatment costs, and survival estimates. This approach will provide insights into how specific assumptions influence cost-effectiveness results, strengthening the robustness of policy recommendations.

### Data sources and Data handling

This study will only use secondary, anonymised data, with no collection of individual-level patient data. All datasets are publicly available or accessed under institutional agreements.

•   NCRI: Aggregated LC incidence, stage, survival data.

•   CSO & Eurobarometer: Demographic and smoking prevalence data.

•   HPO, HIPE, and PCRS: Healthcare cost and resource utilisation data.

## Discussion

This research programme addresses a critical evidence gap in the economic evaluation of LCS in Ireland. By integrating screening eligibility modelling, stage-specific cost analysis, and cost-effectiveness evaluation, it provides a comprehensive assessment of the economic and financial implications of LDCT screening. This is the first study of its kind in Ireland, generating policy-relevant evidence to inform decisions on resource allocation, programme feasibility, and long-term cancer control strategies. The findings will support national healthcare planning and align with the strategic objectives of the National Cancer Control Programme (NCCP).

### Policy implications

The results of this study will provide empirical evidence to guide policymakers in evaluating the feasibility of a national LCS programme. By estimating the size of the high-risk population, the programme will inform financial planning, workforce requirements, and infrastructure investments needed for sustainable implementation. The stage-specific cost analysis will demonstrate the financial impact of late-stage LC care, reinforcing the economic argument for early detection. Furthermore, the cost-effectiveness model will provide a structured framework for assessing alternative screening strategies, allowing policymakers to balance clinical benefits with economic feasibility.

Beyond lung cancer, this research establishes a methodological foundation for future health economic evaluations in Ireland, particularly in oncology and chronic disease screening. The findings may contribute to broader discussions on risk-based screening policies, resource allocation, and long-term healthcare sustainability. Additionally, as real-world data on screening implementation and outcomes become available, the model can be refined and expanded to guide policy adjustments over time

### Strengths and limitations

A key strength of this research programme lies in its comprehensive methodological approach, integrating diverse datasets from the National Cancer Registry Ireland (NCRI), the Hospital In-Patient Enquiry (HIPE) system, the Pharmaceutical Reimbursement Service (PCRS), and the Central Statistics Office (CSO). By leveraging dynamic Markov modelling, discrete event simulation (DES), and cost-effectiveness analysis (CEA), the study ensures robust, policy-relevant estimates. The use of advanced modelling techniques in R enhances the accuracy and transparency of cost-effectiveness calculations, while adherence to international reporting standards, including CHEERS (Consolidated Health Economic Evaluation Reporting Standards), ensures comparability with global research.

Despite these strengths, some limitations must be acknowledged. The screening eligibility analysis relies on Eurobarometer 2017 data for smoking history, as neither the 2022 Census nor the Healthy Ireland survey provides detailed pack-year estimates. While Eurobarometer offers the most comprehensive available data, more recent or Ireland-specific datasets would improve precision.

Another limitation is the availability and granularity of healthcare cost data. While administrative datasets such as HIPE and PCRS provide cost estimates, they lack detailed information on outpatient care, diagnostics, and treatment-specific costs. In the absence of a comprehensive national cost database, this study will estimate certain costs using Diagnosis-Related Groups (DRGs) and published unit costs, which may introduce some uncertainty into cost-effectiveness estimates. Validation through expert consultation, a Delphi survey and sensitivity analyses will help mitigate this issue.

Finally, as with all model-based economic evaluations, assumptions regarding screening uptake, treatment pathways, and long-term health outcomes are necessary. While probabilistic and deterministic sensitivity analyses will be conducted to explore uncertainty, the real-world effectiveness of LCS in Ireland will depend on actual programme implementation, participation rates, and adherence to follow-up care. Future studies should incorporate local pilot screening data to refine these projections.

### Dissemination and knowledge translation

The findings of this research programme will be widely disseminated to maximise impact. Peer-reviewed publications will be produced for each work package, ensuring methodological transparency and academic contribution. Results will also be presented at national and international conferences, facilitating engagement with researchers, clinicians, policymakers, and public health experts.

To support real-world policy translation, an evidence summary will be developed and shared with healthcare stakeholders, including the Department of Health, the NCCP, and the HSE. Where appropriate, findings will be adapted for professional and public engagement through policy briefs, stakeholder workshops, and targeted knowledge-sharing initiatives. In addition, opportunities to communicate key insights through social media and professional networks will be explored to enhance visibility and accessibility of the research.

## Conclusion

This research programme represents a substantial contribution to health economic research in Ireland, providing the first comprehensive economic evaluation of LCS in the country. By integrating modelling of screening eligibility, stage-specific cost analysis, and cost-effectiveness evaluation, it addresses both immediate policy questions and long-term healthcare planning considerations.

## Ethic and consent

This study will be based exclusively on secondary data analysis. All datasets used are either publicly available in anonymised form or were accessed under institutional agreements in compliance with GDPR and data governance requirements.

- Eurobarometer 87.1 (2017): This dataset contains de-identified, individual-level data and is publicly available through the GESIS data archive. Informed consent was obtained from participants at the time of data collection by the European Commission, and ethical oversight was provided by the data collectors in accordance with EU regulations.
- National Cancer Registry Ireland (NCRI), Healthcare Pricing Office (HPO), Hospital In-Patient Enquiry (HIPE), and Pharmaceutical Reimbursement Service (PCRS) data were accessed in aggregated or fully anonymised form under institutional agreements. These datasets do not contain identifiable personal information, and no direct contact with participants occurred.
- CSO Census and Population Projections are fully anonymised and publicly available.

As no new data were collected and all data were anonymised or publicly available, additional ethical approval or participant consent was not required for this study.

## Data Availability

No data are associated with this article.
