# Supplementary material for: Protocol for the economic evaluation of LCS in Ireland: modelling costs, eligibility, and outcomes
Source: HRB Open Res. 2025 Jul 23;8:55. Originally published 2025 Apr 15. [Version 2] doi: 10.12688/hrbopenres.14126.2 (PMC12416482; doi:10.12688/hrbopenres.14126.2)
Supplement: Peer review history

# Reviewer report: Reviewer response for version 2

**DOI:** 10.21956/hrbopenres.15636.r48522

**Published:** 2025-08-30

**Authors:** David Baldwin

**Affiliations:** 1 University of Nottingham, Nottingham, England, UK

I am happy with the responses to the reviewers

Is the study design appropriate for the research question?

Yes

Is the rationale for, and objectives of, the study clearly described?

Yes

Are sufficient details of the methods provided to allow replication by others?

Partly

Are the datasets clearly presented in a useable and accessible format?

Partly

Reviewer Expertise:

Lung cancer screening

I confirm that I have read this submission and believe that I have an appropriate level of expertise to confirm that it is of an acceptable scientific standard.

# Reviewer report: Reviewer response for version 1

**DOI:** 10.21956/hrbopenres.15525.r47388

**Published:** 2025-07-10

**Authors:** Geertruida H de Bock, Danrong Zhong

**Affiliations:** 1 University of Groningen, Groningen, The Netherlands

This is a nice manuscript with a very interesting analysis. Lung cancer screening reduced lung cancer mortality by shifting lung cancer stage distribution. Cost-effectiveness modeling studies can provide critical evidence to inform policymakers and accelerate the implementation of screening programs. I hope these comments contribute to strengthening the manuscript.

 1. What can we learn from this study compared to what already have been published? Is the Irish context different from other countries, like UK? How this is different?

**Work package 1: eligible population for screening**

 1. It’s written that “To ensure accuracy, the Markov model will be calibrated using historical smoking prevalence trends and validated against published Irish smoking data.” This is not clear. What is the difference between the calibration and the validation data set?

 2.. Clarity pack-year threshold

 The analysis focuses on individuals aged 55-74 with a smoking history of 15 or 20 pack-years: which threshold was applied (15 or 20)? For former smokers, is there a cessation time threshold (e.g. quit <10 years or <15years)?

 3.. Reference for LCS recommendation

 The statement ‘the risk group typically recommended for LCS’ , can you added reference on that? Is based on which guidelines? There are several guidelines as well as lung cancer prediction models. Which one is selected and why?

**Work package 2: cost estimation**

 1. Data source

 The listed sources (National Cancer Registry Ireland, Healthcare Pricing Office, etc.) do not include: costs for LDCT scan, follow-up diagnostics (e.g. biopsies for suspicious nodules).

 Prices are very political sensitive. Especially for the expensive treatments (immunotherapy) prices are based on negotiation (often per hospital). Where the expensive drugs will be the main driver in the cost-effectiveness. How the authors will deal with this? How to deal with expected changes in prescription behavior? (e.g. in future immunotherapy will be also prescribed for early stage LC, there will be new immunotherapy drugs in the near future etc). And for immunotherapy, not all of the patients were eligible for this expensive treatment, how to estimate the percentage of immunotherapy eligibles in different stages?

 2. Discount rate

 What discount rate was applied (e.g. 3% or 5%)?

 Rationale for the chosen discount rate.

**Work package 3: Cost-effectiveness analysis of LCS**

 1. Mortality reduction estimate

 ‘Screening effectiveness: mortality reduction estimates from NLST (20%) and NELSON (24%)’, which screening effectiveness are really use in this study?

 The effective of screening is critically dependent on:

 (1) High-risk population definitions: NLST (aged 55-75 ever smokers, more than 30 pack-years and currently smoke or quit less than 15 years), for NELSON (aged 50-75, smoke more than 15 cigarettes per day for more than 25 years, or 10 cigarettes per day for more than 30 years, currently smoke or quit less than 10 years). These differing eligibility criteria may lead to variations in observed mortality reduction. Eligibility of these 2 trials are different from the definition in this protocol (aged 55-74, with pack year 15-20), is it appropriate to apply mortality reduction estimates from NLST/NELSON to high-risk population in this study (aged 55-74, with pack year 15-20)?

 (2) Screening protocol: in NLST, is 3 rounds of the annual screening, for NELSON is 4 rounds of screening (baseline, regular screening after previous round screening with 1-year interval, 2-year interval, and 2.5 year interval). For this protocol, compared screening (annal, biennial and one-time strategies) to no screening, is it appropriate to apply mortality reduction from NLST/NELSON?

 (3) For NELSON, 24% mortality reduction is only report on male, for female, is 33% reduction, will this analysis use 24% for both gender or account for the higher female reduction?

 2. Screening strategies

 The CEA compares LDCT screening to no screening, evaluating annal, biennial and one-time strategies, rationale for selecting these screening strategies.

 3. I miss an impact analysis in WP3. What will be the impact of the implementation of LC screening for the budget, for the personal and how this relates to stop-smoking programs? The Budget impact analysis should specify its time horizon, is a 3-year, 5-year or 10-year timeframe more appropriate for LC screening?

**Model validation**

 Different models ( DES: discrete event simulation model; dynamic Markov-based population model) were used in this protocol, will these models undergo validation? If so:

 1. which independent datasets or published studies will be used for validation?

 2. validation metrics: which outcomes will be compared to assess model accuracy? For example:

 Stage distribution (screen-detected vs. clinical detected cancers)

 Tumor detection rates

 Size distribution of detected tumors…

 Is the rationale for, and objective of, the study clearly described?

 Yes

 Is the study design appropriate for the research question?

 Yes

 Are sufficient details of the methods provided to allow replication by other?

 Partly

 Are the datasets clearly presented in a useable and accessible format?

 Partly

 Competing interests: No competing interests were disclosed.

 Reviewer expertise: lung cancer screening, cost-effectiveness evaluation.

Is the study design appropriate for the research question?

Yes

Is the rationale for, and objectives of, the study clearly described?

Yes

Are sufficient details of the methods provided to allow replication by others?

Partly

Are the datasets clearly presented in a useable and accessible format?

Partly

Reviewer Expertise:

epidemiology, screening, lung cancer

We confirm that we have read this submission and believe that we have an appropriate level of expertise to confirm that it is of an acceptable scientific standard, however we have significant reservations, as outlined above.

# Response

**Published:** 2025-07-17

**Authors:** Tatiana Bezdenezhnykh

**Affiliations:** General Practice, Royal College of Surgeons in Ireland, Dublin, Leinster, Ireland

General comment: 

 We thank the reviewer for their positive feedback and thoughtful suggestions. We appreciate the recognition of the study’s potential to inform national policy and accelerate implementation of lung cancer screening in Ireland. The reviewer has helpfully identified several areas where greater clarity, detail, and justification are needed. We have responded to each point in detail below and will revise the manuscript to incorporate the necessary clarifications and additions.

Work Package 1: Eligible Population for Screening

 1. Calibration and Validation 

 Calibration refers to adjusting model parameters (e.g. smoking transitions) so that simulated outputs align with historical Irish smoking trends and external estimates. For example, we will calibrate smoking transition rates from Eurobarometer against Health Ireland survey smoking prevalence. Validation refers to comparing model outputs, such as future prevalence trends and eligibility estimates against external empirical findings (from Ireland and other jurisdictions: UK, France, Australia)

 2. Pack-year thresholds and cessation 

 The primary analysis will focus on individuals aged 55–74 years with a smoking history of 20 or more pack-years who have quit within the past 15 years, reflecting current Irish eligibility criteria. We will also compare this group to those eligible under other widely used international guidelines, including NELSON, USPSTF 2013, and USPSTF 2021, which define risk groups typically recommended for lung cancer screening.

 3. Reference for risk group selection 

 The definition aligns with USPSTF 2021, NELSON, and NCCP guidance. We will revise the protocol to cite these guidelines and clarify why we selected this risk group: it balances comparability with major trials while reflecting Irish population risk profiles.

Work Package 2 – Cost Estimation

 We appreciate the reviewer’s comments and agree that estimating accurate costs, especially for diagnostics and high-cost therapies, is a key challenge, particularly in the Irish context. The points below are clarified in the updated protocol.

 1. LDCT scans and follow-up diagnostics: Ireland does not have unit costs for individual outpatient procedures like LDCT or biopsy. Instead, costs are bundled into DRGs under the AR-DRG system per case. We will use average DRG-based costs from the HIPE system to estimate diagnostic costs, supplemented by external sources (e.g. estimates available from National Screening Service Ireland) and tested in sensitivity analysis.

 2. High-cost drugs and immunotherapy: We will base our estimates on NCRI data on treatment patterns by stage, alongside published drug prices. The proportion of patients eligible for immunotherapy at each stage will be estimated using NCRI treatment data and supported by published evidence on PD-L1 expression and clinical criteria.

 3. Future changes and uncertainty: We recognise that treatment guidelines and prescribing patterns will evolve. To account for this, we will include scenario analyses to explore the impact of expanded indications and new therapies over time. 

 4. Discount Rate

 We apply a 4% discount rate for both costs and outcomes (in the cost-effectiveness analysis), in line with HIQA guidance.

Work Package 3 – Cost-Effectiveness Analysis

 1. Mortality reduction estimates 

 We agree that trial-based mortality reductions (e.g. NLST 20%, NELSON 24–33%) are specific to their designs and populations. We further clarify in the updated version of the protocol that we do not directly apply these reductions. Instead, screening benefit is modelled through a stage-shift approach, with survival based on stage and detection mode (e.g. screen-detected vs. clinical), consistent with the UK NSC’s ENaBL model.

 2. Rationale for screening strategies 

 We selected annual, biennial, and one-time screening to mirror strategies evaluated in NLST, NELSON, and UK pilot studies. These reflect realistic programme options for Ireland and enable trade-off analysis between frequency, benefit, and cost.

 3. Budget impact and implementation 

 We agree that this is an important and policy-relevant extension of the analysis. While our primary focus is on the economic evaluation, which lays the foundation for a BIA, we recognise the relevance of implementation considerations. As such, factors such as workforce capacity, scanner availability, and integration with smoking cessation services will be discussed outside of the scope of this project.

Model validation

 We agree that validation is an essential part of the modelling process. Our protocol involves three distinct models, which will undergo validation appropriate to their structure and intended purpose.

 1. Validation datasets: 

 For the costing model, which is based on NCRI data, we will validate internal consistency (e.g. cost by stage aligns with expected treatment pathways) and cross-reference cost estimates with external published costing studies (e.g. from UK and EU contexts).

 For the CEA model, we will validate model outputs against both Irish and international data: 

- NCRI data: LC incidence, stage distribution, survival by stage. 
- Published trial data: NLST and NELSON outcomes (e.g. stage shift, detection rates). 
- UKLS/TLHC data: where relevant, to validate outputs related to screening effectiveness. 

2. We will assess the external validity of model outputs using the following metrics, where data availability allows: 

- Stage distribution of lung cancers: among screen-detected vs. symptomatically diagnosed cases. Compare how NCRI data (only clinical cases) aligns with NLST, NELSON, and UKLS data (e.g. % Stage I in NLST = 52%, in TLHC ≈ 62%). 

- Detection rate: Number of cancers detected per 1,000 screened individuals; benchmark against published trial and pilot data (NLST, NELSON, TLHC).

- Screening uptake and eligibility proportions: % of population eligible under different criteria; % of eligible individuals screened. Cross-validated with Irish data and UK pilot uptake figures. The Irish LC screening pilot is underway at its early stages. If the data is available early, it will be certainly used in validation. 

- Mortality outcomes (indirectly): modelled lung cancer mortality reductions compared to NLST (20%), NELSON (24%–33%) under similar screening protocols (as a sense check for reasonableness). 

- Survival outcomes by stage and detection mode: Check alignment with NCRI.

- Overdiagnosis estimates: Compare % of cases estimated to be overdiagnosed in the model to published ranges (e.g. NLST estimate ≈ 18–25%).

# Reviewer report: Reviewer response for version 1

**DOI:** 10.21956/hrbopenres.15525.r47383

**Published:** 2025-06-03

**Authors:** Stephen Wade

**Affiliations:** 1 The University of Sydney, Sydney, Australia

The article describes a protocol for an economic evaluation of a lung cancer screening (LCS) in Ireland. The goals of the analysis are to obtain estimates of 1) the eligible population (as the screening is targeted), 2) the stage-specific costs of lung cancer care, and 3) the incremental cost-effectiveness of screening versus no-screening.

 Overall, much of the protocol reflects existing practise; however there are such enormous differences in published estimates of 1) and 3) above that it brings in to question the reliability of "existing practise" and whether this is sufficient to support good decision making in Ireland (or other contexts).

 With reservations, the protocol is sound. I have a few comments about methods.

**Estimating the Eligible Population for Screening**

 Data-driven approaches are preferable to Markov models which have stricter assumptions about smoking behaviour and how that behaviour is going to be reported by participants. In Australia, for example, a model-based approach produced estimates that differed by a factor of two from data-driven methods (see "National Lung Cancer Screening Program – Updated participation modelling" https://www.health.gov.au/resources/publications/nlcsp-participation-modelling, and see https://doi.org/10.17061/phrp34342410 for the data-driven approach). This is a large discrepancy!

 The budget impact is already subject to great uncertainty because of the difficulty in estimating participation rate; having a faulty denominator would be a terrible start.

 Unless absolutely necessary, I think developing a model to determine who attends screening in 2057 does not seem like a useful - or even scientifically valid - exercise.

**Estimating Stage-Specific Costs of LC Care**

 ICIs and targeted therapies are only administered when a specific subtype is detected (and often only reimbursed when the disease is at a specific stage). Ideally, then, some estimate of the prevalence of the relevant subtype (by stage) would be used to inform the cost. I did not find a reference to this in the protocol. The cBioportal has been used in the past to inform the costing of metastatic NSCLC - this would likely be a useful template for costing here (see Ngo et al https://doi.org/10.1007/s40273-023-01291-6).

**Cost-Effectiveness Analysis (CEA) of LCS**

 I note that the model intended as a basis (Snowsill 2018) produced vastly different results from the more recent 2022 evaluation supplied to the UK National Screening Committee (see https://view-health-screening-recommendations.service.gov.uk/lung-cancer/). 

 From a review published in 2022: "The range of cost estimates in the literature is broad, from US$1464 to $2 million per quality-adjusted life year (QALY) gained depending on the setting, modelling approach, and policy question." (see https://doi.org/10.1016/S0140-6736(22)01694-4). There is significant (catastrophic) uncertainty that renders these analyses of low value in answering the question of whether LCS is cost-effective compared to no screening. *Even within jurisdiction, with shared data sources, a factor of three difference can be observed in estimate of benefit* (see Figure 4 of "Evaluation of the Benefits and Harms of Lung Cancer Screening With Low-Dose Computed Tomography: Modeling Study for the US Preventive Services Task Force" https://doi.org/10.1001/jama.2021.1077).

 The collection of evidence (all based on modelling) does not convince me that, say, a biennial program is more (or less) cost-effective than an annual program; yet this is still a frequently tested hypothesis. Similarly, there are long-standing questions about pack-year versus risk-model based criteria - frequently tested but no clear answer.

 There is no data source or methodological leap presented in this protocol that convinces me that this study will prove different to the others. Perhaps a stronger focus should be placed here on *sensitivity* of the cost-effectiveness to parameters or *Ireland-specific* policy questions; with the acknowledgement that these models might not be able to answer "if" something is cost-effective, but they might identify which factors are most significant to future investigations (or pilots) and policy design.

Is the study design appropriate for the research question?

Partly

Is the rationale for, and objectives of, the study clearly described?

Yes

Are sufficient details of the methods provided to allow replication by others?

Partly

Are the datasets clearly presented in a useable and accessible format?

Not applicable

Reviewer Expertise:

bayesian statistics, health economics, cancer epidemiology, tobacco control

I confirm that I have read this submission and believe that I have an appropriate level of expertise to confirm that it is of an acceptable scientific standard, however I have significant reservations, as outlined above.

## References

[1] : Estimates of the eligible population for Australia’s targeted National Lung Cancer Screening Program, 2025–2030. *Public Health Research and Practice* .2024;35(1) : 10.17061/phrp34342410 10.17061/phrp34342410 40443073

[2] : Development and Validation of txSim: A Model of Advanced Lung Cancer Treatment in Australia. *PharmacoEconomics* .2023;41(11) : 10.1007/s40273-023-01291-6 1525-1537 10.1007/s40273-023-01291-6 PMC1057019737357233

[3] : Lung cancer screening. *The Lancet* .2023;401(10374) : 10.1016/S0140-6736(22)01694-4 390-408 10.1016/S0140-6736(22)01694-4 36563698

[4] : Evaluation of the Benefits and Harms of Lung Cancer Screening With Low-Dose Computed Tomography. *JAMA* .2021;325(10) : 10.1001/jama.2021.1077 10.1001/jama.2021.1077 PMC920891233687469

# Response

**Published:** 2025-07-17

**Authors:** Tatiana Bezdenezhnykh

**Affiliations:** General Practice, Royal College of Surgeons in Ireland, Dublin, Leinster, Ireland

General comment: 

 We appreciate the reviewer’s insight into methodological uncertainty and agree that while modelling remains a useful tool, its limitations must be carefully acknowledged. Variation in model estimates comes about, in part, from different modelling assumptions. We believe that establishing a domestic model for Ireland is an important part of capacity building. While current practice certainly is imperfect, we feel it is important that we at least establish a baseline modelling capacity up which we can further develop and refine.

1. Thank you for highlighting the discrepancy observed between model-based and data-driven approaches in the Australian context. The point is well made and it does give us pause for thought regarding the value of simulation vs empirical analysis. Our analysis is anchored on data-derived baseline eligibility estimates and reflects a plausible scenario for future smoking behaviours in Ireland. We believe that modelling is useful in understanding why anticipated and actual participation might differ. Moreover, while modelling might not accurately anticipate the level of actual participation, it hopefully can at least be useful in predicting trends in eligibility over time. Indeed, where eligibility determined by age and smoking history, such trends are arguably quite predictable. That said, significant uncertainties regarding participation will naturally persist. While we have chosen a Markov model to simulate mid-term trends in screening eligibility, we acknowledge the importance of grounding this in real-world data. To address potential discrepancies between modelling approaches, we will validate our Markov-based estimates with those derived from the data-driven method, as suggested by the reviewer. Finally, we agree that projecting as far as 2057 adds limited value given the uncertainty involved and is of modest current policy relevance. Accordingly, we have revised the projection horizon to 2045. 
2. Thank you for this useful suggestion. We acknowledge the importance of this point, especially when therapies can differ so much in terms of their cost. We will include estimates of histologic subtypes and relevant biomarker prevalence (e.g. EGFR, ALK) by disease stage, informed by international datasets such as cBioPortal , if local registry data (NCRI) on assignment of targeted therapies is not enough or unavailable. We will also revise our costing methodology to weight targeted therapy uptake accordingly. 
3. We acknowledge the point the reviewer makes regarding the heterogeneity of estimates and the questions this raises regarding the value of simulation evidence. We would make some observations in reply, some of which relate to replies above. One is that there are often good reasons for variations in simulation estimates, often down to fundamental differences in the way that models are constructed, but also to more easily observable differences in the way strategies are simulated and compared. For this reason, the variation in CEA estimates may be less than initially apparent. That said, it is undeniable that estimates vary, leaving decision makers with considerable uncertainty.  We do not believe that such uncertainty is reason to abandon CEA modelling. The process of modelling construction and testing allows the modeller to appreciate what are the important factors that influence CEA estimates, where additional evidence is needed and where models should be refined. Ireland currently does not have a domestic cancer screening modelling platform, applied to lung screening or otherwise. Establishing this as a capability is important, not just for value for money assessment, but also for budget impact analysis and capacity planning.  We do need to frankly admit the limitations of modelling and communicate the uncertainties to decision makers. We feel we would be in a better position to do so with a model than without one, despite the inherent flaws of such approaches.

# Reviewer report: Reviewer response for version 1

**DOI:** 10.21956/hrbopenres.15525.r46935

**Published:** 2025-05-13

**Authors:** David Baldwin

**Affiliations:** 1 University of Nottingham, Nottingham, England, UK

The protocol paper describes the plans for an economic review of lung cancer screening in Ireland. This is an important topic as lung cancer screening has the potential to save many lives.

 My comments reflect the experience gained from the health economics analysis undertaken for the UK National Screening Committee.   I appreciate that some of the comments will present considerable difficulty but failure to address some aspects risk getting the wrong results - as indeed the paper you quote by Snowshill et al presented.  Note that the ICER changed some 18 fold once the correct input variables were included.  It is therefore important that the latest report, and not the 2018 paper is both read by the authors and quoted in their protocol.  It is available here please scroll down to the final report link (2022). https://view-health-screening-recommendations.service.gov.uk/lung-cancer/

 1. Defining the high risk group and participation.  The authors intend to attempt to select on the basis of pack-years using Eurobarometer.  This is entirely dependent on the accuracy of those data so that needs to be confirmed.  I would suggest that it would be better to model both eligibility and participation using the English real-world data which is unlikely to be very different in Ireland. This should at least be checked again the Eurobarometer and a sensitivity analysis using the English data done.  This would be first to calculate the number of ever smokers in the eligible age range, then model a 50% response to the invite then approximately 50% of these being eligible and most of these having a CT. - 20-25% of the initial invite.  We are seeing improved participation as the programme rolls out but this would be a far better way to estimate unless Eurobarometer is super accurate.

 2. The NCRI data is being used to measure stage distribution and survival - how accurate is this.  Does it contain performance status?  The latter is important if you are to model the effect of a screen because studies show that most people are PS 0-1 in screening in contrast to clinically detected.  This translates to better treatment outcomes which means survival is better even accounting for lead time effect.  If you are going to adjust for overdiagnosis then you should also include this, otherwise you underestimate the impact of screening.  Interval cancers should be modelled as clinically detected.  Incidence round cancers will have a greater proportion of early stage than prevalence. 

 3. I was confused about the use of mortality derived from NLST and NELSON and then references to stage specific survival.  Which are you using?  The intention to control for over diagnosis  yet use mortality from the studies (which controls for  overdiagnosis, lead and length time implies two seperate analyses?

 4. The intention to use LYG and discounts is fine but there will need to be adjustment for the very different fitness levels that we see between screen detected and clinically detected.  For the English analysis we were able to use survival by stage and performance status to show the impact in real world data of the screen - according to whatever stage was detected.  Studies both show the proportion in each stage and the proportion in each PS category. We also knew survival by stage in the screening cohort (UKLS).

 5. what stage distribution are you using for screening detected lung cancer. NLST seems to have detected 10 % points fewer stage 1 than the English data ( 52 vs 62).  The UKNSC analysis was via a natural history model that modelled NLST data so likely underestimates the value of screening.

 6. incidental findings are a key issue - in the English analysis we simply added a cost derived from the paper by Bartlett et al, but this does not add any benefit, which we now see (in the working programmes) is evident.

 I hope these comments have been helpful

Is the study design appropriate for the research question?

Yes

Is the rationale for, and objectives of, the study clearly described?

Yes

Are sufficient details of the methods provided to allow replication by others?

Partly

Are the datasets clearly presented in a useable and accessible format?

Partly

Reviewer Expertise:

Lung cancer screening

I confirm that I have read this submission and believe that I have an appropriate level of expertise to confirm that it is of an acceptable scientific standard, however I have significant reservations, as outlined above.

# Response

**Published:** 2025-07-17

**Authors:** Tatiana Bezdenezhnykh

**Affiliations:** General Practice, Royal College of Surgeons in Ireland, Dublin, Leinster, Ireland

General Comment: 

 We thank the reviewer for their thoughtful and detailed feedback, drawing on extensive experience with the UK National Screening Committee’s work. The comments raised are insightful and will help refine the protocol to ensure greater methodological robustness and relevance to real-world policy contexts. We note the reviewer’s comments about the importance of using the most up to date iteration of the Snowsill et al work, thus, we will adjust the methodology to align it with 2022 NCS report.  

1. We appreciate this recommendation. We will use Eurobarometer data only to inform smoking histories to estimate the proportion of eligible people. Eurobarometer data is closely aligned with Irish Census 2022 smoking prevalence and is the only source known to us that reports smoking length and the average number of cigarettes for the Irish quitters. We will further apply UK real-world data to estimate the potential uptake. (e.g., 50% response rate to invitation and 50% of responders meeting criteria). To support the base case methodology, we will provide estimates using the approach suggested above. 
2. Thank you for this suggestion. The initial protocol indeed did not give consideration as to how health status would vary between those with screen or clinically detected cancers and what implications this might have for survival. We see that performance status (PS) could be one useful way for differentiating health status and prognosis between such individuals. PS is unfortunately not currently captured in the NCRI dataset. We are likely to incorporate such effects by assuming survival probabilities that differ by the mode of presentation, if we can adequately support this with data from either Irish hospital data or screening cohorts such as NLST or UKLS. 
3. We thank the reviewer for highlighting this important clarification. We acknowledge that using both trial-derived mortality reductions and stage-specific survival curves is not a plausible approach. We have updated the protocol to clarify this modelling approach and avoid confusion.  In line with the 2022 ENaBL model, our model will simulate disease progression through preclinical and clinical stages and rely on stage-specific survival estimates from NCRI and UK sources. The benefit of screening will be estimated via stage shift, i.e. the change in stage distribution due to screening, and heterogeneity in survival for clinically-detected and screen-detected cancers.   
4. Thank you for raising this important point. We fully agree that performance status (PS) is a key determinant of survival and treatment eligibility, and that screen-detected patients are generally fitter than those diagnosed clinically. Unfortunately, performance status is not currently captured in the NCRI dataset, which limits our ability to directly model PS-specific survival using Irish data. To address this, we plan to follow the approach taken in other economic evaluations, including the updated analysis of the UK NSC’s ENaBL model. 
5. Thank you for this helpful observation. As our model structure is based on the ENaBL model, which itself was calibrated using NLST data, we will retain these underlying assumptions for consistency. However, we recognise that this may underestimate the potential benefits of screening, particularly in terms of early-stage detection. We will explicitly acknowledge this limitation in the protocol and, where feasible, explore the impact of varying stage distributions in sensitivity analysis to assess the robustness of our results 
6. We agree that incidental findings are a relevant consideration in the cost-effectiveness analysis. As in the English analysis, we plan to include an estimate of the cost of follow-up investigations for incidental findings (e.g., from Bartlett et al.), but recognise that doing so without capturing potential downstream benefits introduces an inconsistency. We acknowledge that a complete evaluation would ideally account for both the costs and potential health benefits of incidental findings. However, given current evidence limitations and the scope of this analysis, quantifying these benefits is beyond what we can reasonably include at this stage. We will clearly note this as a limitation in the protocol and highlight it as an area for future refinement as more real-world data emerge from screening programmes. 
